# First evidence of anticoagulant rodenticides in fish and suspended particulate matter: spatial and temporal distribution in German freshwater aquatic systems

**DOI:** 10.1007/s11356-018-1385-8

**Published:** 2018-03-01

**Authors:** Matthias Kotthoff, Heinz Rüdel, Heinrich Jürling, Kevin Severin, Stephan Hennecke, Anton Friesen, Jan Koschorreck

**Affiliations:** 1 Department Environmental and Food Analysis, Fraunhofer Institute for Molecular Biology and Applied Ecology IME, Auf dem Aberg 1, 57392 Schmallenberg, Germany; 2 Department Environmental Specimen Bank and Elemental Analysis, Fraunhofer Institute for Molecular Biology and Applied Ecology IME, Auf dem Aberg 1, 57392 Schmallenberg, Germany; 3 German Environment Agency (Umweltbundesamt), 06813 Dessau-Rosslau, Germany

**Keywords:** Anticoagulant rodenticides, Environmental monitoring, High-resolution mass spectrometry, Bream, Suspended particulate matter, Environmental Specimen Bank, Biocides

## Abstract

Anticoagulant rodenticides (ARs) have been used for decades for rodent control worldwide. Research on the exposure of the environment and accumulation of these active substances in biota has been focused on terrestrial food webs, but few data are available on the impact of ARs on aquatic systems and water organisms. To fill this gap, we analyzed liver samples of bream (*Abramis brama*) and co-located suspended particulate matter (SPM) from the German Environmental Specimen Bank (ESB). An appropriate method was developed for the determination of eight different ARs, including first- and second-generation ARs, in fish liver and SPM. Applying this method to bream liver samples from 17 and 18 sampling locations of the years 2011 and 2015, respectively, five ARs were found at levels above limits of quantifications (LOQs, 0.2 to 2 μg kg^−1^). For 2015, brodifacoum was detected in 88% of the samples with a maximum concentration of 12.5 μg kg^−1^. Moreover, difenacoum, bromadiolone, difethialone, and flocoumafen were detected in some samples above LOQ. In contrast, no first generation AR was detected in the ESB samples. In SPM, only bromadiolone could be detected in 56% of the samples at levels up to 9.24 μg kg^−1^. A temporal trend analysis of bream liver from two sampling locations over a period of up to 23 years revealed a significant trend for brodifacoum at one of the sampling locations.

**Electronic supplementary material:**

The online version of this article (10.1007/s11356-018-1385-8) contains supplementary material, which is available to authorized users.

## Introduction

Since the introduction of warfarin as the first anticoagulant rodenticide on the US market in the late 1940s, rodent control worldwide has relied increasingly upon the use of these chemicals. As of 2017, anticoagulant rodenticides constitute more than 95% of the authorized rodenticides as biocides in the European Union (ECHA 2017b). The discovery of anticoagulant rodenticides (ARs) is today recognized as the most important step towards safer and more effective rodent control (Buckle and Eason 2015).

ARs comprise active substances belonging either to the class of 4-hydroxycoumarines such as warfarin or to 1,3-indandione derivatives such as chlorophacinone. Without regard to their chemical structure, ARs are grouped by their ability to prevent blood clotting (coagulation) by the inhibition of vitamin K which is essential for the production of several blood clotting factors such as prothrombin. Typical symptoms of AR intoxication, i.e., internal and external hemorrhages due to the increased permeability of blood vessels, occur several days after consumption of the rodenticide bait. This delayed mode of action is key to the effectiveness of ARs as it overcomes the bait shyness of rats.

Anticoagulant rodenticides are usually divided into first- and second-generation anticoagulant rodenticides (FGARs/SGARs) depending on the date of their introduction on the market. FGARs (i.e., warfarin, chlorophacinon, coumatetralyl) were firstly used in the late 1940s, 1950s, and 1960s, while SGARs (i.e., bromadiolone, difenacoum, brodifacoum, difethialone, flocoumafen) were developed in the 1970s and 1980s following an increasing concern about warfarin-resistant rodents. Ever since, ARs have been extensively used as pesticides to reduce human and animal infections by rodent-borne diseases, for crop protection against voles, or for species conservation on oceanic islands (Masuda et al. 2015). They are nowadays regulated in the European Union (EU) under the Biocidal Products Regulation (EU) No. 528/2012 (BPR) and the Plant Protection Products Regulation (EC) No. 1107/2009 (PPPR), depending on their intended use to either protect human health, animal health and materials or plants and plant products. Both regulations foresee that ARs need to be authorized prior to being made available on the European market. Under the PPPR, difenacoum (Reg. (EU) No. 540/2011) and bromadiolone (Reg. (EU) No. 540/2011) are the only anticoagulant active substances which are approved for the use in plant protection products in the EU. Under the BPR, the approval of eight anticoagulants, i.e., warfarin, chlorophacinone, coumatetralyl, bromadiolone, difenacoum, brodifacoum, difethialone, and flocoumafen as active substances for the use in rodenticides, have just recently been renewed. While the last authorization of an anticoagulant rodenticide as a plant protection product in Germany has expired in 2015, their authorizations as biocides in Germany have recently been prolonged (BVL 2017). As of September 2017, 704 rodenticide products were authorized in Germany under the BPR, of which about 91% contained an anticoagulant active substance, of these 12.2% FGAR and 79.0% SGAR (compare Table 1) (BAuA 2017).

**Table 1:** Current numbers of registered biocidal products in Germany

Active substance	Number of registered products	%
Aluminum phosphide	9	1.3
Brodifacoum	196	27.8
Bromadiolone	127	18.0
Chloralose	51	7.2
Chlorophacinone	14	2.0
Coumatetralyl	14	2.0
Difenacoum	199	28.3
Difenacoum; bromadiolone	4	0.6
Difethialone	26	3.7
Flocoumafen	4	0.6
Hydrogen cyanide	1	0.1
Carbon dioxide	1	0.1
Warfarin	58	8.2
Total	704	100
FGARs	86	12.2
SGARs	556	79.0
Non-ARs	62	8.8

The environmental risk assessment of ARs under the BPR authorization in the EU revealed high risks of primary and secondary poisoning for non-target organisms, which either feed directly on the bait or consume poisoned rodents. Moreover, all SGARs have been identified as being either persistent, bioaccumulative, and toxic (PBT-substances) or very persistent and very toxic (vPvB-substances). These inherent substance properties in combination with the given exposure of non-target organisms via primary and secondary poisoning and the extensive and widespread use of ARs are significant drivers for the likewise widespread contamination of various wildlife species worldwide. It is thus not surprising that residues of anticoagulant rodenticides, especially of the second-generation compounds, have been detected in a large variety of species. Residues of rodenticides were detected for example, in barn owls (Geduhn et al. 2016, Newton et al. 1990), tawny owls (Walker et al. 2008), common buzzards (Berny et al. 1997), golden eagles (Langford et al. 2013), polecats/mink (Elmeros et al. 2018, Fournier-Chambrillon et al. 2004, Ruiz-Suarez et al. 2014, 2016, Shore et al. 2003), weasels (McDonald et al. 1998), stoats (Elmeros et al. 2011), foxes (Berny et al. 1997, Geduhn et al. 2015, McMillin et al. 2008, Tosh et al. 2011), hedgehogs (Dowding et al. 2010), and snails (Alomar et al. 2018).

Most of these environmental monitoring studies focused on the terrestrial compartment, e.g., predatory birds (Gomez-Ramirez et al. 2014, Ruiz-Suarez et al. 2014, Stansley et al. 2014, Thomas et al. 2011) and mammals (Quinn et al. 2012), as well as various non-target rodents (Elliott et al. 2014, Geduhn et al. 2014). However, little to nothing is known so far, about the exposure of aquatic life to ARs and the accumulation of ARs in aquatic food webs.

The environmental exposure assessment within the authorization of anticoagulant rodenticides under the BPR is based on the Emission Scenario Document (ESD) (Larsen 2003) which considers four main scenarios for the application of ARs, i.e., the application in and around buildings, in open areas (in rate holes), at waste dumps, and in the sewer system. Significant releases to surface water bodies are only assumed to occur from the application of ARs in the latter area of use, i.e., in sewer systems. It has been shown that AR can enter sewage treatment plants (STPs) and thereafter contribute to the loads of anticoagulants to receiving surface waters with effluents (Gomez-Canela et al. 2014). A maximum release to the sewerage system and consequently to surface water could result directly from the application of rodent bait into manholes of the sewer system and indirectly from the target animals’ urine, feces, and dead bodies. The application of rodenticides in rainwater sewers which as a rule are not connected to a sewage treatment plant and discharge directly into receiving waters can be considered another release pathway.

Environmental monitoring of AR provides some specific challenges to the investigator. AR can enter the environment via different exposure routes where they have been shown to exhibit acute toxic effects at concentrations in the ppm and ppb range (e.g., bromadiolone (Eason et al. 2002, Thomas et al. 2011): LC_50_ of 2.86 mg L^−1^ for fish, *Lepomis macrochirus*; LD_50_ of 0.56 mg kg^−1^ in rat (oral) (ECHA 2010), or difethialone (ECHA 2007): EC_50_ of 4.4 μg L^−1^ for *Daphnia magna* acute, or LC_50_ of 51 μg L^−1^ for *Oncorhynchus mykiss*. SGARs in particular exhibit a high lipophilicity and environmental persistence and may thus enrich in predator tissues with high fat contents, e.g., mammalian liver (Eason et al. 2002, Thomas et al. 2011), which are complex matrices and thus require elaborate and challenging sample preparation. Furthermore, there are numerous AR substances that may enter the environment and so a comprehensive assessment of the presence of AR requires very sensitive and accurate multi-methods, covering a wide range of different ARs. Several analytical approaches for multi-methods for the quantitative determination of AR in biological samples have been developed, such as liquid chromatography (LC) and also ion chromatography (IC) coupled to tandem mass spectrometry (MS/MS) (Bidny et al. 2015, Chen et al. 2009, Jin et al. 2009, Jin et al. 2008, Marek and Koskinen 2007), two-dimensional LC coupled to MS/MS (Marsalek et al. 2015), IC coupled to fluorescence detection (Jin et al. 2007), methods using high resolution MS (Schaff and Montgomery 2013), and some other strategies that are presented in a review by Imran et al. (2015). Available analytical methods are so far hampered by the number of captured AR, as well as high limits of detection caused by complex biological and environmental matrices that are in contrast to low relevant environmental concentrations. However, the method we apply here is in good agreement (Hernandez et al. 2013) or better (Vandenbroucke et al. 2008a, Vandenbroucke et al. 2008b, Zhu et al. 2013) in terms of number of analytes covered and sensitivity with other LC-MS/MS-based methods for solid biological tissues such as liver and hair.

Good insight is available on risks of AR towards non-target mammals as well as exposure and associated risks of various predators (Christensen et al. 2012, Geduhn et al. 2016, Gomez-Ramirez et al. 2014, Hughes et al. 2013, Langford et al. 2013, Nogeire et al. 2015, Proulx and MacKenzie 2012, Rattner et al. 2014, 2015, Ruiz-Suarez et al. 2014, Thomas et al. 2011).

Even if concentrations are assumed to be low after systematic or accidental exposure of aquatic systems (Fisher et al. 2012, Primus et al. 2005), the environmental impact may yet be relevant due to the high bioaccumulation potential, especially of SGARs (Masuda et al. 2015). So far, no studies are available on AR residues and accumulation in fish or distribution of AR in natural aquatic systems. The aim of this study was to assess the exposure of freshwater fish to anticoagulant rodenticides by analyzing levels of anticoagulants in fish tissues. For this purpose, a highly sensitive and specific multi-method was developed to determine eight anticoagulants, which have been approved under the BPR for the use in rodenticides within the EU (cf. Table 2). We the applied this method in a spatial monitoring study for two time points for fish liver and one for suspended particulate matter (SPM) samples of the German Environmental Specimen Bank (ESB). Finally, retrospective analysis was performed for SPM and fish samples from selected sites to detect time trends. In addition, selected liver samples from otters (*Lutra lutra*) were analyzed to characterize the bioaccumulation potential of ARs in fish-eating mammal species.

**Table 2:** List of AR covered in this study used for quantification and information on used reference standards

AR	AR-generation	Chemical class (derivative)	Purchased from	Purity (%)
Brodifacoum	2	Hydroxycoumarine	Sigma-Aldrich	99.4
Bromadiolone	2	Hydroxycoumarine	Sigma-Aldrich	93.6
Chlorophacinone	1	Indandione	Sigma-Aldrich	98.9
Coumatetralyl	1	Hydroxycoumarine	Sigma-Aldrich	99.9
Difenacoum	2	Hydroxycoumarine	Sigma-Aldrich	98.9
Difethialone	2	Thiocoumarine	Dr. Ehrenstorfer	99.0
Flocoumafen	2	Hydroxycoumarine	Dr. Ehrenstorfer	98.0
Warfarin	1	Hydroxycoumarine	Sigma-Aldrich	≥ 98

## Materials and methods

### Collection and storage of samples

All samples were retrieved from the archive of the German Environmental Specimen Bank.

Bream (*Abramis brama*) samples were analyzed from 17 and 18 sampling locations for 2011 and 2015, respectively, and from 10 sampling years for two specific sampling locations. Sampling locations included 16 riverine sites and one (2011) and two (2015) lakes. Samples were processed and stored according to a dedicated ESB standard operating procedures (SOP) by Klein et al. (2012). SPM was analyzed from the 16 riverine sampling sites sampled in 2015. SPM was collected and processed according to a specific SOP by Ricking et al. (2012).

Otter samples originate from the Upper Lusatia area in the east of Germany (partly Elbe catchment) and represent individuals that died as a result of traffic accidents or lethal diseases. The liver samples of five otter individuals were prepared and analyzed according to the protocol for bream liver samples.

### Method summary

In order to optimize and merge available methods, and to secure the specificity of the method, the rodenticide analysis was performed on a UHPLC-chromatographic unit coupled to a high-resolution mass spectrometer operating at a resolution of 35,000. The adopted method mainly based on Thomas et al. (2011) for fish liver could be used to determine a total of eight different target molecules, as given in Table 2 and Table 3. The specificity of the method is assured by measuring the accurate mass of the analytes in tandem mass spectrometric mode (MS/MS) (see Table 3).

**Table 3:** Accurate masses of ion transitions of rodenticides as used for the multiple monitoring method. The Q-Exactive instrument was run at a resolution of 35,000 ± 10 ppm

Substance	Theoretical mass of precursor [m/z]	Captured mass of product 1 [m/z]	Captured mass of product 2 [m/z]
Flocoumafen	541.16322	161.02353	289.08545
Bromadiolone	525.0707	250.06194	n.d.
Brodifacoum	521.07578	135.04408	187.03854
Difenacoum	443.16527	135.04442	293.13202
Warfarin	307.09758	161.02234	250.06195
Chlorophacinone	373.0637	145.02859	201.04637
Coumatetralyl	291.10267	141.07021	247.11263
Difethialone	537.05294	151.02104	n.d.

Table 2 harbors information on the reference substances used for quantification. Stable isotopically labeled (deuterated) internal standards (IS) were only available for bromadiolone (as D5; Campro Scientific, Germany, 99% D, 95% chemical, Lot # AB126P2), warfarin (as D5; Campro Scientific, Germany, 99% D, 99% chemical, Lot # E305P28), and chlorophacinone (as D4; Chiron AS, Norway, 99.4% D, 99% chemical Lot # 14266). The IS were added to the samples, but not used for evaluation in the final method.

A sample of about 0.5 g fish matrix (liver or muscle; frozen, cryo-milled ESB material) is mixed with roughly 3.5 g Na_2_SO_4_ (ratio 1:7), 100 μL IS solution (three IS, each 100 ng mL^−1^), and 5 mL acetone in a 15-mL polypropylene test tube. This mixture is treated for 30 min in an ultra-sonic bath and for the same time on a vortex shaker. Subsequently, the test tube is centrifuged at 4000 rpm for 5 min. The clear supernatant is forwarded to a fresh test tube, whereas the pellet is extracted with an additional 4 mL volume of fresh acetone. The combined extracts were mixed with 1 mL of diethyl ether and evaporated in a N_2_-stream at 50 °C to dryness. The remaining extract was then dissolved in 1 mL methanol and homogeneously mixed by treating for 5 min in an ultra-sonic bath. The slightly turbid suspension was forwarded to a 1.5-mL tube and centrifuged at 15,000 rpm for 2 min. The cleared supernatant was finally filtrated through a 25-mm diameter, 0.45-μm regenerated cellulose (RC) type membrane filter, before filling into a UHPLC (ultra-high performance liquid chromatography) vial for analysis.

A minimum of two solvent-based blank samples (up to four) were analyzed in every measurement series. Matrix-based quality control samples containing the rodenticides in defined concentrations (adapted to the expected concentration range in the samples: here 1.4 and 14 μg kg^−1^ for otter and SPM, and 1.0 and 10 μg kg^−1^ for bream liver) were measured about every 15 samples. Suitable rodenticide free matrices were identified in a preliminary screening. All samples were measured at least in duplicate, as specified in the respective table captions.

### Instrumental parameters

A UHPLC Acquity (Waters), coupled to an Orbitrap Q-Exactive Plus (Thermo Scientific) high-resolution mass spectrometer, run in the multiple reaction monitoring mode with electrospray negative (ES-) ionization was used for all chemical analyses. Accurate masses of parent and daughter ions, as well as MS-parameters, were according to Table 3. The used column was 100 × 2 mm BEH C18, 1.7 μm (Waters), the column temperature was 55 °C, and 20 μL sample volume was injected and run with a flow of 0.35 mL min^−1^. The solvents used were A: methanol +2 mM ammonium acetate in water (5 + 95, *v*/*v*) and B: methanol containing 2 mM ammonium acetate. The used UHPLC gradient program was 0 min 100% A → 10 min 100% B → 13 min 100% B → 15 min 100% A. Under the given conditions, of bromadiolone, two diastereomeric partners elute, which are reported here as a sum.

### Method development and method validation

Initially, a comparison of AR concentrations in bream liver and fillet was performed by applying a crude preliminary method that had not been optimized. For difethialone and brodifacoum, we found 100- or 80-fold higher concentrations in liver, respectively. So, it was decided to focus on bream liver samples for further method development and subsequent analysis of environmental samples.

Commercial stable isotope labeled standards were purchased to improve the method. After repeated measurement cycles and calibrations, however, it was found that the analytical parameters are much better when using an external matrix matched calibration. So, the final method does not use the signals for the IS, but an external matrix calibration.

Calibration and validation of the method were performed by standard addition techniques using matrix calibrations in the range from 0.02 to 20.0 μg kg^−1^ and were evaluated to the lowest calibration level within the linear range of a calibration. Each calibration solution contained 100 μL of IS solution which were spiked with 25 μg L^−1^, resulting in 5 μg kg^−1^ of each IS. The handling and measurement of the calibration and validation samples were identical to the treatment of the test samples. For validation of the method, six bream liver and SPM samples of 0.5 g each were fortified with defined AR at individual limit of quantification (LOQ) concentrations to prove for accuracy, repeatability, and precision at the LOQ level (standard addition technique), according to Table 4. Each sample was fortified with 100 μL of a solution containing all rodenticides in the respective concentrations ranging from 0.1 to 100 μg L^−1^ and with 100 μL of the IS solution. Otter liver was used to generate a respective matrix calibration, but due to limited sample material, no separate otter liver validation could be performed.

**Table 4:** Studied AR and respective analytical parameters of method validation by fortification of respective matrix, *n* = 6

Substance	Bream liver			SPM		
	LOQ level [μg kg^−1^]	Recovery [%]	RSD [%]	LOQ level [μg kg^−1^]	Recovery [%]	RSD [%]
Flocoumafen	0.2	100	5.4	1.0	98	6.9
Bromadiolone	2.0	95	8.1	1.0	96	4.8
Brodifacoum	1.0	93	6.6	2.0	98	5.9
Difenacoum	0.2	96	9.7	1.0	98	10.3
Warfarin	0.2	103	6.9	0.2	102	7.5
Chlorophacinone	1.0	93	7.2	2.0	116	26.3
Coumatetralyl	0.2	110	4.1	0.2	106	5.4
Difethialone	1.0	95	3.3	1.4	103	4.6

Due to varying sensitivities of individual AR, the dynamic ranges of the calibrations are different, but none of them showed an exponential behavior. To keep the procedure constant, even after the decision to omit using the IS, their addition to the samples was continued. For the given calibration ranges, all functions were linear and show coefficients of determination (*r*^2^) of at least 0.99. The validated limits of quantification (LOQ) and standard deviations (SD) are given in Table 4. All data are reported on a wet weight basis.

Both matrices, bream liver and SPM, could be successfully validated at the indicated LOQ levels. These levels range in a substance, but also in a matrix-dependent manner from 0.2 to 2.0 μg kg^−1^, and reflect the lowest achievable values according to observations derived from the matrix calibration functions shown in Fig. S1 of the Electronic supplementary material (ESM). The recoveries are within 90–110% and the relative standard deviation (RSD) is ≤ 10% (*n* = 6). The only exception is chlorophacinone whose mean recovery is 116% and RSD 26.3% in SPM. This seems acceptable since no quantitative data are being reported for chlorophacinone in this study. The achieved LOQs are similar or lower than recently published LC-MS/MS-based multi-methods for AR in tissues, ranging from 0.9 to 250 μg kg^−1^ (Fourel et al. 2017a, Hernandez et al. 2013, Jin et al. 2009, Marek and Koskinen 2007, Marsalek et al. 2015, Smith et al. 2017, Vandenbroucke et al. 2008b).

### Analysis of temporal trends

Temporal trends for brodifacoum in fish tissue (wet weight data) were analyzed by applying a software tool from the German Environment Agency (LOESS-Trend, Version 1.1, based on Microsoft Excel). The application fits a locally weighted scatterplot smoother (LOESS) with a fixed window width of 7 years through the annual rodenticide levels. Then, tests on the significance of linear and non-linear trend components are conducted by means of an analysis of variance (ANOVA) following the procedure of Fryer and Nicholson (1999). For years with analytical results less than the LOQ, the data gaps were treated as ½ LOQ values.

## Results and discussion

### Results of environmental analysis

#### Spatial comparison: measurement of samples from different ESB sampling sites of the years 2011 and 2015

**Fig. 1:**
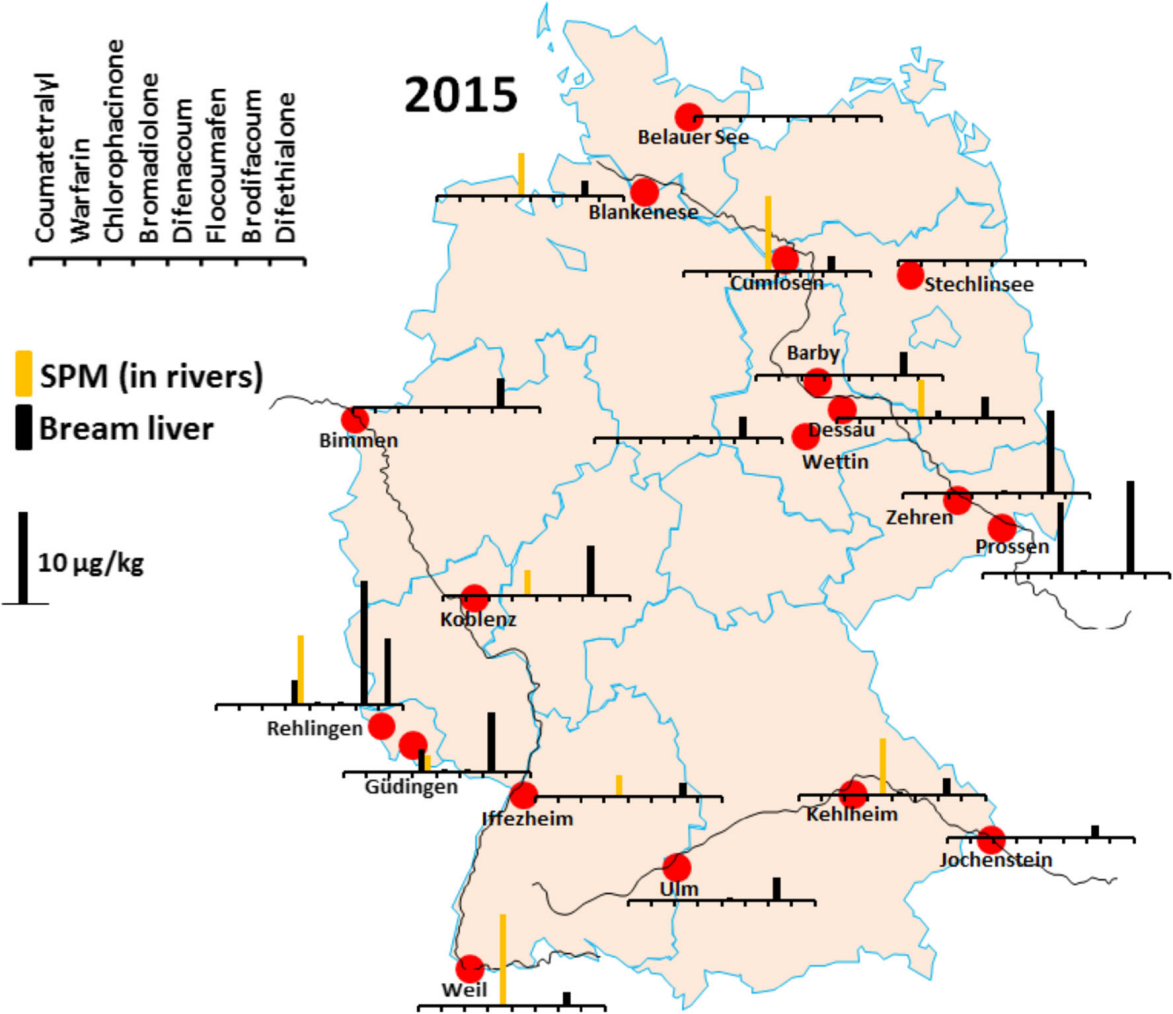
Overview of 18 bream and 16 SPM sampling sites. Results of the spatial analysis for eight ARs in bream liver and SPM are displayed as black and yellow bars, respectively. For detailed results, see Table S1 and S3

Results of the spatial monitoring exercise are presented in Table S1 (2011) and Table S6 (2015) in the electronic supplemental material and are summarized in Fig. 1 (for 2015) along with the spatial distribution of the sampling sites across Germany. Within all bream liver samples, only SGARs were found above the LOQ. For the year 2015, brodifacoum was the major AR found. It was detected in 88% of the samples with a maximum concentration of 12.5 μg kg^−1^ (average (Ø) 3.4 μg kg^−1^ and median 2.1 μg kg^−1^). Difenacoum was found in 44% of the samples at comparably lower concentrations of up to 0.7 μg kg^−1^ (Ø 0.1 μg kg^−1^). Bromadiolone was found in 17% of the samples at peaks of 7.1 μg kg^−1^ with Ø of 0.6 μg kg^−1^, difethialone in 6% with highest levels at 6.3 μg kg^−1^, and flocoumafen in 12% at highest levels of 0.3 μg kg^−1^. For 2011, quite different substance and concentration patterns were found, as presented in Table S1, which may be due to the seasonal character of substance usage and varying intervals between application and sampling. To our knowledge, this is the first evidence of AR in freshwater fish tissue.

In addition, a set of co-located suspended particulate matter (SPM) samples from the year 2015 were analyzed. The results are presented in Table S3 and also included in Fig. 1. In contrast to results of the bream liver samples, in SPM, only bromadiolone was found above LOQ in 56% of the 16 samples with highest values of 9.2 μg kg^−1^ (Ø 4.9 μg kg^−1^; median 4.3 μg kg^−1^).

No ARs were detected > the LOQ in the five otter livers that were analyzed in addition to bream and SPM to exemplary include a fish-eating mammal as a top predator in the food web in this study. In contrast, in a study using French otter samples from 2010, 10% of the tested otter samples were contaminated with bromadiolone at levels of 400 and 850 μg kg^−1^ fresh weight (Lemarchand et al. 2010).

#### Temporal trend analysis: retrospective monitoring for rodenticides in fish liver samples from Saar River/Rehlingen and Elbe River/Prossen

Based on the results of the 2011 and 2015 spatial analysis, the sampling sites in Rehlingen at the Saar River and Prossen at the Elbe River were chosen for the temporal analysis.

The results of the temporal analysis are summarized in S4 (Saar/Rehlingen) and Table S5 (Elbe/Prossen).

**Fig. 2:**
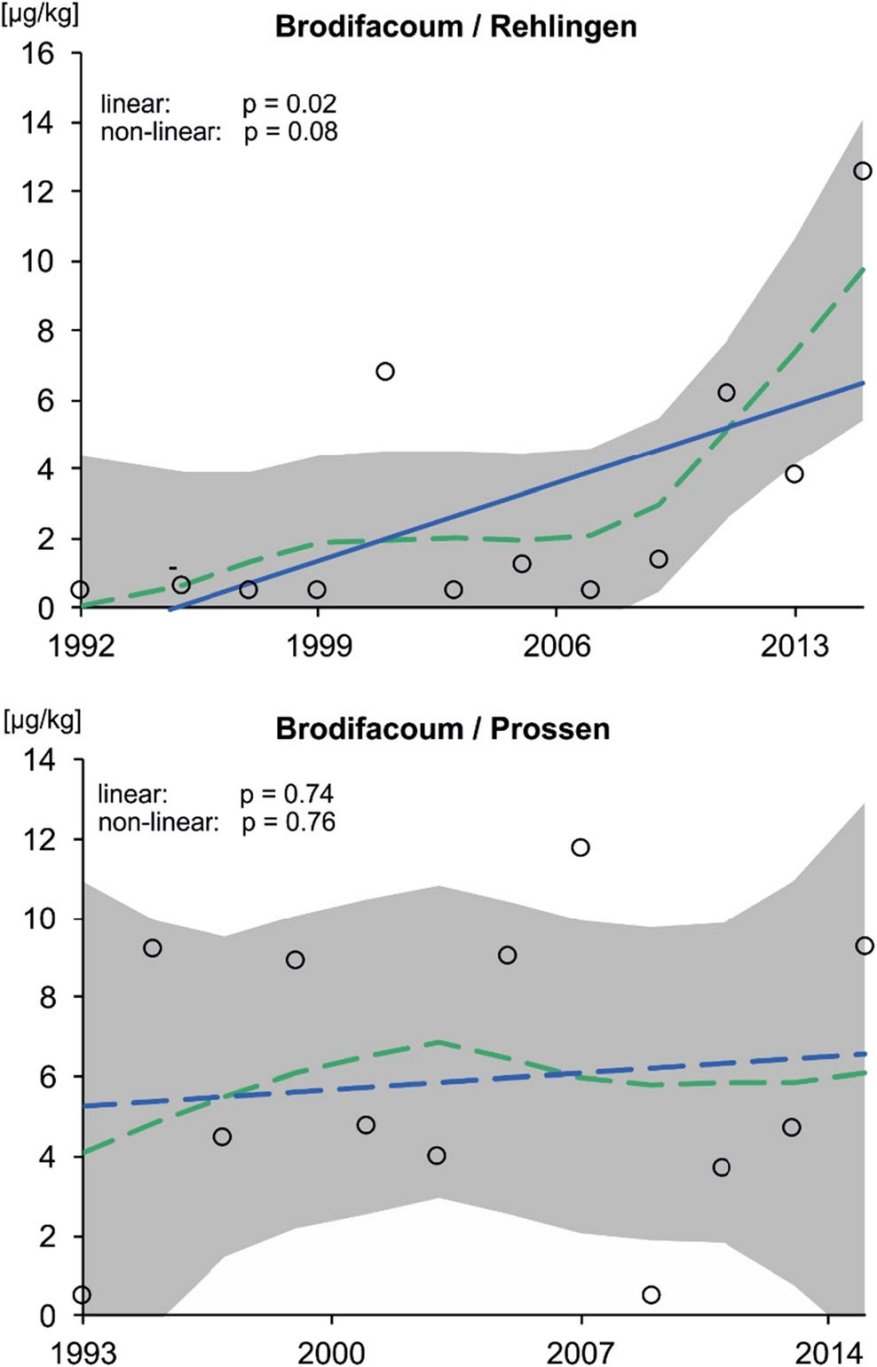
Time trend analysis of both sampling sites for brodifacoum using the LOESS-Trend tool (compare “Materials and methods” section). Circles reflect actual results (mean values of replicates of pooled fish samples), while the blue solid or dashed line reflects the linear fit, the green dashed line the dynamic fit, and the gray area the confidence interval (α = 0.05). For mean value calculations, data below the LOQ were substituted by a concentration of 50% of the LOQ (LOQ = 1.0 μg kg^−1^ for brodifacoum; compare Table S4 and S5 in the ESM). “-” indicates values results below LOQ

From these temporal data, a significant time trend could be drawn only for brodifacoum at Saar/Rehlingen (Fig. 2). This trend indicated an average increase of brodifacoum at 0.3 μg kg^−1^ per year for the observed period and 1.3 μg kg^−1^ per year for the last 7 years.

Notably, brodifacoum was the most abundant AR measured in fish from both locations. Concentrations ranged between about 1 and 13 μg kg^−1^ in fish from Rehlingen and between 4 and 12 μg kg^−1^ in fish from Prossen, where it was found below LOQ only in the years 1992 and 2009. At Rehlingen also, bromadiolone, difenacoum, flocoumafen, and difethialone were found occasionally and at comparably low levels. Interestingly, for both sampling sites, the diversity of detected AR was higher in 2015 than in the years before. SPM was not subject to a retrospective analysis.

#### Assessment of relevance of rodenticide residues in fish and SPM

The analysis of fish samples at the different ESB sampling sites revealed the detectable occurrence in the order brodifacoum, difenacoum, bromadiolone, difethialone, and flocoumafen at levels above the LOQ. In contrast, in SPM, only bromadiolone was detectable.

Only SGARs were found above LOQ of the ARs measured in this study. This could be related to the higher persistency and potential for bioaccumulation of SGAR in comparison to FGAR. The partition coefficients n-octanol/water (log K_ow_), as a measure for lipophilicity and bioaccumulation potential, for FGARs are < 5 (at environmentally relevant pH) while the respective values for SGARs are all > 5 (at environmentally relevant pH). Also, available toxicity studies with rats show much shorter half-lives of FGAR in livers when compared to SGAR which may indicate faster elimination rates in target and non-target organisms (Daniels 2013). Another plausible reason for the lack of FGARs in the analyzed fish samples might be that FGARs are generally used less frequently than SGARs, especially for the control of rats in sewer systems, which is assumed to be the source of emissions to surface water bodies. A survey of 508 local municipal authorities in Germany responsible for the rat control in sewers (Krüger and Solas 2010) indicated that bromadiolone followed by difenacoum and brodifacoum were used most often by local authorities for the control of brown rats in sewer systems.

#### Comparison with bioconcentration factors, adsorption coefficients, and use patterns

The bioconcentration factors (BCF, the ratio of a substance concentration in water and in fish tissue and expressed as L kg^−1^) of fish as stated in the respective public Assessment Reports for their approval under BPR decrease in the following order: difethialone (39,974; estimated), brodifacoum (35,645; estimated), flocoumafen (24,300; measured), bromadiolone (460; measured), chlorophacinone (22.75; estimated), warfarin (≤ 21.6; measured), coumatetralyl (11.4; measured) (ECHA 2017a). The BCF values may explain why SGARs were detectable in the ESB fish samples, while FGARs were not. The organic carbon adsorption coefficients K_oc_ [L kg^−1^], as given in the respective Assessment Reports for each of the active substances, increase in the order of warfarin (174), coumatetralyl (258), brodifacoum (9155), bromadiolone (14,770), chlorophacinone (75,800), flocoumafen (101,648), difenacoum (1.8* 10^6^), difethialone (about 10^8^) (ECHA 2017a). According to this, other highly adsorptive anticoagulants such as difenacoum, which is according to Krüger and Solas (2010) commonly used for sewer baiting in Germany, should also be expected to absorb to SPM (assumed that comparable amounts are emitted). There may be several reasons why this is not the case: SPM samples in the ESB archive were pooled samples of 12 monthly sub-samples, whereas only one ESB fish sample was collected per year after spawning at each of the riverine sampling sites. Depending on a seasonal exposure, higher or lower findings, compared to the concentrations actually found in this study, may be expected in SPM (e.g., when exposed in spring after treatment campaigns in municipal rodent control, AR can be expected in SPM), but the occurrence in fish that are sampled in a different season compared to the treatment might be unlikely, especially for FGAR with a low bioaccumulation potential. However, this cannot fully explain the exclusive presence of bromadiolone and we are unclear why other ARs were not detected in this matrix.

The varying treatment lengths, intervals, and substance patterns of AR treatment campaigns in Germany may also help to explain the occasional detections of other SGAR, as their presence may reflect the major AR applied in the catchment that year. Data on the amounts of AR that were used are unfortunately rarely available (Pohl et al. 2015). Rodenticides, which have been used most often by municipal authorities for sewer baiting (Krüger and Solas 2010), were those found most frequently in fish (difenacoum, brodifacoum, and bromadiolone) and bromadiolone in SPM.

Rough estimations suggest that the AR concentrations detected in fish are plausible given the available data on concentrations in sewage treatment plant (STP) effluent (Gomez-Canela et al. 2014) and the known BCF of the detected compounds in fish (For details, see ESM).

An important aspect of rodenticides was recently identified to be the metabolism by Fourel et al. (2017b). They found a high abundance of trans-bromadiolone in red kite, indicating individual metabolic rates for the two bromadiolone enantiomers. If fish could also metabolize bromadiolone isomers selectively, this could explain why we found bromadiolone more frequently in SPM compared to fish liver. This does, in turn, not help to understand why other ARs were not found in SPM.

#### Synopsis

In summary, our findings demonstrate that contamination of wildlife with anticoagulant rodenticides, especially SGARs, also involves aquatic species and is not confined to predatory birds or mammals of the terrestrial food web. We detected residues of SGARs in fish samples from almost every ESB sampling site, including the rivers Rhine, Elbe, and Danube. The ubiquitous exposure of fish is in contrast to the rather low concentrations of SGARs in biocidal products which ranged from 25 mg kg^−1^ (difethialone) to 75 mg kg^−1^ (difenacoum). An amount of approximately 50 kg of anticoagulant rodenticide active substance is used annually for rat control in sewers and above ground by municipal authorities in Germany, with approximately 75% were used exclusively for sewer baiting (Krüger and Solas 2010). Given this relatively moderate amount of use, the prevalence of detectable rodenticide residues in fish samples appears surprisingly high. Whether this is entirely accounted for by the persistent and bioaccumulative properties of the SGARs requires investigation. In general, there remains a lack of understanding about both the impacts of rodenticides on aquatic life and the pathways by which these compounds enter the environment. There are few published data on rodenticide levels in waste water (Gomez-Canela et al. 2014) or surface water and no information on what specific substances or amounts are used. Experimentally derived BCF values for ARs are not always available and modeled BCF value may not enable a sound assessment of the potential for bioaccumulation in fish. Therefore, it is important to generate a better overview on the temporal spatial occurrence of AR in freshwater environments and to identify relevant sources and entry pathways. Further research is needed to unravel the exposure of freshwater environments to rodenticides. This may involve environmental fate studies as well as additional spatial and temporal monitoring activities. Monitoring of AR can thereby provide additional key information for their environmental risk assessment and the need to set appropriate risk mitigation measures within their authorization as biocides in the European Union.

## Electronic supplementary material

ESM 1 (DOCX 187 kb)
