# Supplementary material for: First evidence of anticoagulant rodenticides in fish and suspended particulate matter: spatial and temporal distribution in German freshwater aquatic systems
Source: Environ Sci Pollut Res Int. 2018 Mar 1;26(8):7315–25. doi: 10.1007/s11356-018-1385-8 (PMC6447514; doi:10.1007/s11356-018-1385-8)
Supplement: Supplementary file 1 — (DOCX 187 kb) [file 11356_2018_1385_MOESM1_ESM.docx]

**Supplementary Material**

**First Evidence of Anticoagulant Rodenticides in Fish and Suspended Particulate Matter: Spatial and Temporal Distribution in German Freshwater Aquatic Systems**

Matthias Kotthoff^1^, Heinz Rüdel^2^, Heinrich Jürling^1^, Kevin Severin^1^, Stephan Hennecke^1^, Anton Friesen^3^, Jan Koschorreck^3^

^1^Fraunhofer Institute for Molecular Biology and Applied Ecology IME, Department Environmental and Food Analysis, Auf dem Aberg 1, 57392 Schmallenberg, Germany

^2^Fraunhofer Institute for Molecular Biology and Applied Ecology IME, Department Environmental Specimen Bank and Elemental Analysis, Auf dem Aberg 1, 57392 Schmallenberg, Germany

^3^German Environment Agency (Umweltbundesamt), 06813 Dessau-Rosslau, Germany

# **Plausibility Check of Tissue Concentrations**

## **Description of Calculations**

For the plausibility check of the results, an estimation of fish tissue concentrations from sewage treatment plant (STP) effluent concentrations was made by applying the bioconcentration factor (BCF). Gómez-Canela et al. (Gomez-Canela et al. 2014) reported levels of rodenticides in Spanish STP effluents. The following formula (2) relates the water concentration (C_water_ in µg L^-1^) of a compound to the fish tissue concentration (C_fish_ in µg kg^-1^ wet weight): (1) Definition: BCF = C_fish_ / C_water_ (BCF unit: L kg^-1^) (2) C_fish_ = BCF * C_water_ (unit: µg kg^-1^ wet weight). Additionally, the following assumptions were made: Assumption 1: a dilution of 10 is assumed for the STP effluent in the receiving waters (European_Chemicals_Bureau 2003): (3) C_water_ = C_effluent_ / 10 (unit: µg L^-1^). Assumption 2: it is assumed that lipophilic rodenticides accumulate in the lipid fraction of tissues; a factor of 2 was applied to consider the higher fat content of liver in comparison to whole fish (about 10 % in ESB bream liver samples, about 5 % in laboratory fish tests which were used to develop estimation models for BCF): (4) C_fish_ = C_fishliver_ / 2 (unit: µg kg^-1^ wet weight); Equations (2) and (4) were combined and converted to: (5) C_fishliver_ = BCF * C_water_ * 2 (unit: µg kg^-1^ wet weight). Combined with equation (3) the complete formula thus reads: (6) C_fishliver_ = BCF * (C_effluent_ / 10) * 2 (unit: µg kg^-1^ wet weight). Example calculation: BCF for brodifacoum: 36,134 L/kg (ECHA 2010a); brodifacoum effluent concentration: 38.4 ng L^-1^ which corresponds to 0.04 µg L^-1^ (rounded value). (7) C_fishliver_ = 36,134 * (0.04 / 10) * 2 = 289 µg kg^-1^ wet weight.

## **Estimation of fish tissue concentrations from literature-retrieved STP effluent concentrations**

In the EU assessment reports for brodifacoum (ECHA 2010a), bromadiolone (ECHA 2010b) and difethialone (ECHA 2007) BCF values are estimated according to QSAR equations given in the Technical Guidance Document (European_Chemicals_Bureau 2003) on the base of experimental K_OW_. The respective calculated BCF are 36,134 for brodifacoum, 575 and 339 for bromadiolone (575 chosen for further comparisons) and 39,974 for difethialone.

Gomez-Canela et al. reported AR levels in Spanish STP effluents (Gomez-Canela et al. 2014). Ranges of concentrations in effluents were < 5 ng L^-1^ to about 60 ng L^-1^ for bromadiolone and < 12 ng L^-1^ to about 40 ng L^-1^ for brodifacoum. Difethialone analysis was not covered in that study. By applying the above mentioned BCF, theoretical fish tissue concentrations can be calculated from this data. Additionally, the following assumptions were made: 1, a dilution of 10 was assumed for the STP effluent in the receiving waters (European_Chemicals_Bureau 2003); 2, a factor of 2 was applied to consider the higher fat content of liver in comparison to whole fish (see section 1.1 Description of Calculations).

Using the above data as input, the following maximum fish liver concentrations were calculated: brodifacoum, 289 µg kg^-1^; bromadiolone, 6.9 µg kg^-1^ (wet weight related). The quantified concentrations of up to about 13 µg kg^-1^ wet weight for brodifacoum (Tables 5, 6, 8, and 9 in the main paper) are clearly lower (factor of about 22) than the levels estimated from the Spanish STP effluent data. For bromadiolone (Tables 5, 6, 8, and 9 in the main paper), on the other hand, the estimated levels are well in the range of the concentrations of up to about 7 µg kg^-1^ wet weight in fish liver. Under the assumption that the STP effluent levels from Spain are comparable to those in Germany and that degradation is not relevant for these persistent compounds, the detected concentration levels in fish liver seem plausible at least for bromadiolone.

# **Calibration Functions**


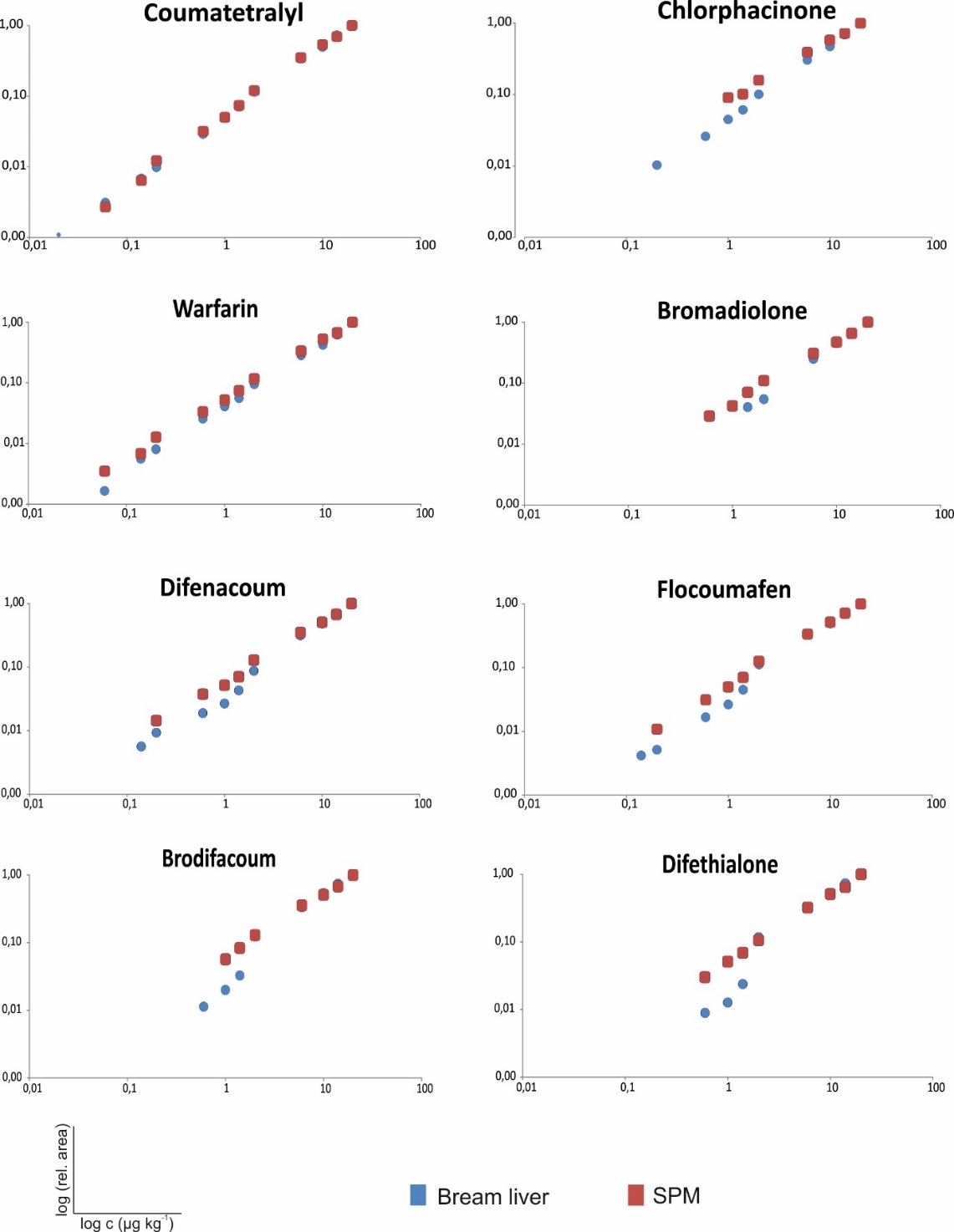


Figure S1: Collection of matrix calibration lines for all analytes in bream muscle (blue spots) and SPM (red spots). To visualize the full range of calibration, the axes are both given in logarithmic units. The x axis reflects the analyte concentration in µg kg^-1^ sample matter. The y axis reflects normalized area units (max^-1^).

# Detailed Results

Table S1: Overview of the results of the analyzed rodenticides in bream liver samples for 2011. Results reflect mean ± standard deviation (SD), n=3 (wet weight data). Only those rodenticides with positive detections above LOQ (compare Table 4) are displayed. Shaded lines: data of sites selected for trend monitoring. Values in brackets indicate only one or two individual results above LOQ, in order to calculate the mean, ½ LOQ was used in this case.

| Sampling location (river, site) | Bromadiolone | | Difenacoum | | Flocoumafen | | Brodifacoum | | Difethialone | |
| --- | --- | --- | --- | --- | --- | --- | --- | --- | --- | --- |
|  | Mean Concentration ± SD [µg kg^-1^] | | | | | | | | | |
| Elbe, Prossen | < LOQ |  | < LOQ |  | < LOQ |  | 3.67 | ± 1.10 | < LOQ |  |
| Elbe, Zehren | < LOQ |  | < LOQ |  | < LOQ |  | < LOQ |  | < LOQ |  |
| Elbe, Barby | < LOQ |  | < LOQ |  | < LOQ |  | 1.24 | ± 0.14 | < LOQ |  |
| Elbe, Cumlosen | < LOQ |  | < LOQ |  | < LOQ |  | (0.76) |  | < LOQ |  |
| Elbe, Blankenese | < LOQ |  | < LOQ |  | < LOQ |  | (0.79) |  | < LOQ |  |
| Saale, Wettin | < LOQ |  | < LOQ |  | < LOQ |  | < LOQ |  | < LOQ |  |
| Mulde, Dessau | < LOQ |  | < LOQ |  | < LOQ |  | < LOQ |  | < LOQ |  |
| Danube, Ulm | < LOQ |  | < LOQ |  | < LOQ |  | < LOQ |  | < LOQ |  |
| Danube, Kelheim | < LOQ |  | < LOQ |  | < LOQ |  | < LOQ |  | < LOQ |  |
| Danube, Jochenstein | < LOQ |  | < LOQ |  | < LOQ |  | < LOQ |  | < LOQ |  |
| Rhine, Weil | < LOQ |  | < LOQ |  | < LOQ |  | < LOQ |  | < LOQ |  |
| Rhine, Iffezheim | < LOQ |  | < LOQ |  | < LOQ |  | < LOQ |  | < LOQ |  |
| Rhine, Koblenz | < LOQ |  | < LOQ |  | < LOQ |  | 1.95 | ± 0.15 | < LOQ |  |
| Rhine, Bimmen | < LOQ |  | < LOQ |  | < LOQ |  | (0.76) |  | < LOQ |  |
| Saar, Güdingen | < LOQ |  | < LOQ |  | < LOQ |  | (0.86) |  | < LOQ |  |
| Saar, Rehlingen | < LOQ |  | < LOQ |  | < LOQ |  | 6.22 | ± 2.13 | 2.13 |  |
| Lake Belau | < LOQ |  | < LOQ |  | < LOQ |  | < LOQ |  | < LOQ |  |
|  |  |  |  |  |  |  |  |  |  |  |

Table S2: Overview of the results of the analyzed rodenticides in bream liver samples for 2015. Results reflect mean ± standard deviation (SD), n=3 (wet weight data). Only those rodenticides with positive detections above LOQ (compare Table 4) are displayed. Shaded lines: data of sites selected for trend monitoring.

| Sampling location (river, site) | Bromadiolone | | Difenacoum | | Flocoumafen | | Brodifacoum | | Difethialone | |
| --- | --- | --- | --- | --- | --- | --- | --- | --- | --- | --- |
|  | Mean Concentration ± SD [ µg kg^-1^] | | | | | | | | | |
| Elbe, Prossen | 7.05 | ± 0.15 | 0.34 | ± 0.03 | < LOQ |  | 9.24 | ± 0.38 | < LOQ |  |
| Elbe, Zehren | < LOQ |  | 0.26 | ± 0.01 | < LOQ |  | 8.34 | ± 0.34 | < LOQ |  |
| Elbe, Barby | < LOQ |  | < LOQ |  | < LOQ |  | 2.28 | ± 0.18 | < LOQ |  |
| Elbe, Cumlosen | < LOQ |  | < LOQ |  | < LOQ |  | 1.53 | ± 0.02 | < LOQ |  |
| Elbe, Blankenese | < LOQ |  | < LOQ |  | < LOQ |  | 1.49 | ± 0.11 | < LOQ |  |
| Saale, Wettin | < LOQ |  | 0.23 | ± 0.01 | < LOQ |  | 2.12 | ± 0.21 | < LOQ |  |
| Mulde, Dessau | < LOQ |  | 0.69 | ± 0.01 | < LOQ |  | 2.05 | ± 0.21 | < LOQ |  |
| Danube, Ulm | < LOQ |  | 0.23 | ± < 0.01 | < LOQ |  | 2.22 | ± 0.19 | < LOQ |  |
| Danube, Kelheim | < LOQ |  | 0.21 | ± 0.01 | < LOQ |  | 1.70 | ± 0.19 | < LOQ |  |
| Danube, Jochenstein | < LOQ |  | < LOQ |  | < LOQ |  | 1.26 | ± 0.04 | < LOQ |  |
| Rhine, Weil | < LOQ |  | < LOQ |  | < LOQ |  | 1.35 | ± 0.09 | < LOQ |  |
| Rhine, Iffezheim | < LOQ |  | < LOQ |  | < LOQ |  | 1.32 | ± 0.10 | < LOQ |  |
| Rhine, Koblenz | < LOQ |  | < LOQ |  | < LOQ |  | 5.10 | ± 0.16 | < LOQ |  |
| Rhine, Bimmen | < LOQ |  | < LOQ |  | < LOQ |  | 2.95 | ± 0.26 | < LOQ |  |
| Saar, Güdingen | 2.21 | ± 0.26 | 0.28 | ± 0.01 | 0.21 | ± < 0.01 | 5.97 | ± 0.12 | < LOQ |  |
| Saar, Rehlingen | 2.36 | ± 0.18 | 0.28 | ± 0.01 | 0.29 | ± 0.01 | 12.54 | ± 1.22 | 6.63 | ± 0.65 |
| Belauer See | < LOQ |  | < LOQ |  | < LOQ |  | < LOQ |  | < LOQ |  |
| Stechlin See | < LOQ |  | < LOQ |  | < LOQ |  | < LOQ |  | < LOQ |  |

Table S3: Overview of the results of the analyzed rodenticides in SPM samples for 2015. Results reflect mean ± standard deviation (SD), n=3 (wet weight data). Rodenticides with detections below LOQ (compare Table 4) are not displayed.

| Sampling location (river, site) | Bromadiolone | |
| --- | --- | --- |
|  | Mean Concentration ± SD [µg kg^-1^] | |
| Elbe, Prossen | 6.92 | ± 0.09 |
| Elbe, Zehren | < LOQ |  |
| Elbe, Barby | < LOQ |  |
| Elbe, Cumlosen | 3.77 | ± 0.18 |
| Elbe, Blankenese | 7.56 | ± 1.10 |
| Saale, Wettin | < LOQ |  |
| Mulde, Dessau | 1.63 | ± 0.44 |
| Danube, Ulm | 9.24 | ± 0.45 |
| Danube, Kelheim | 2.66 | ± 0.12 |
| Danube, Jochenstein | 5.70 | ± 0.93 |
| Rhine, Weil | < LOQ |  |
| Rhine, Iffezheim | < LOQ |  |
| Rhine, Koblenz | < LOQ |  |
| Rhine, Bimmen | 4.33 | ± 0.27 |
| Saar, Güdingen | 2.10 | ± 0.16 |
| Saar, Rehlingen | < LOQ |  |

Table 8: Data of retrospective monitoring in samples of bream liver from the river Saar near Rehlingen; wet weight data, n = 4.

Values in brackets indicate only one or two individual results above LOQ, in order to calculate the mean, ½ LOQ was used in this case.

^#^ These data were taken from Table 5 and Table 6 (no additional analysis).

| Sampling Year | Bromadiolone | | Difenacoum | | Flocoumafen | | Brodifacoum | | Difethialone | |
| --- | --- | --- | --- | --- | --- | --- | --- | --- | --- | --- |
|  | **mean + standard deviation* [µg kg^-1^ bream liver]** | | | | | | | | | |
| 1992 | < LOQ |  | < LOQ |  | < LOQ |  | < LOQ |  | < LOQ |  |
| 1995 | < LOQ |  | < LOQ |  | < LOQ |  | (0.66) |  | < LOQ |  |
| 1997 | 2.03 | ± 0.27 | < LOQ |  | < LOQ |  | < LOQ |  | < LOQ |  |
| 1999 | < LOQ |  | < LOQ |  | < LOQ |  | < LOQ |  | < LOQ |  |
| 2001 | < LOQ |  | 0.43 | ± 0.14 | < LOQ |  | 6.82 | ± 2.62 | < LOQ |  |
| 2003 | < LOQ |  | < LOQ |  | < LOQ |  | < LOQ |  | < LOQ |  |
| 2005 | < LOQ |  | < LOQ |  | < LOQ |  | 1.23 | ± 0.13 | < LOQ |  |
| 2007 | < LOQ |  | < LOQ |  | < LOQ |  | < LOQ |  | < LOQ |  |
| 2009 | < LOQ |  | 0.25 | ± 0.06 | < LOQ |  | 1.40 | ± 0.33 | < LOQ |  |
| 2011^#^ | < LOQ |  | < LOQ |  | < LOQ |  | 6.22 | ± 2.13 | < LOQ |  |
| 2013 | < LOQ |  | 0.65 | ± 0.12 | < LOQ |  | 3.81 | ± 0.82 | < LOQ |  |
| 2015^#^ | 2.36 | ± 0.18 | 0.28 | ± 0.01 | 0.29 | ± 0.01 | 12.54 | ± 1.22 | 6.63 | ± 0.65 |

Table 9: Data of retrospective monitoring in samples of bream liver from the river Elbe near Prossen; wet weight data, n = 4.

* for mean value calculations data below the LOQ were substituted by a concentration of 50 % of the LOQ (LOQ = 0.2 µg kg^-1^ to 2.0 µg kg^-1^ for all compounds; compare Table 4). – results < LOQ.
^#^ These data were taken from Table 5 and Tale 6 (no additional analysis).

| Sampling Year | Bromadiolone | | Difenacoum | | Flocoumafen | | Brodifacoum | | Difethialone | |
| --- | --- | --- | --- | --- | --- | --- | --- | --- | --- | --- |
|  | **mean + standard deviation* [µg kg^-1^ bream liver]** | | | | | | | | | |
| 1993 | < LOQ |  | < LOQ |  | < LOQ |  | < LOQ |  | < LOQ |  |
| 1995 | < LOQ |  | < LOQ |  | < LOQ |  | 9.23 | ± 0.74 | < LOQ |  |
| 1997 | < LOQ |  | < LOQ |  | < LOQ |  | 4.48 | ± 0.68 | < LOQ |  |
| 1999 | < LOQ |  | < LOQ |  | < LOQ |  | 8.89 | ± 2.83 | < LOQ |  |
| 2001 | < LOQ |  | < LOQ |  | < LOQ |  | 4.74 | ± 1.55 | < LOQ |  |
| 2003 | < LOQ |  | < LOQ |  | < LOQ |  | 3.98 | ± 2.31 | < LOQ |  |
| 2005 | < LOQ |  | < LOQ |  | < LOQ |  | 9.04 | ± 1.67 | 1,03 |  |
| 2007 | < LOQ |  | < LOQ |  | < LOQ |  | 11.78 | ± 1.23 | < LOQ |  |
| 2009 | < LOQ |  | < LOQ |  | < LOQ |  | < LOQ |  | < LOQ |  |
| 2011^#^ | < LOQ |  | < LOQ |  | < LOQ |  | 3.67 | ± 1.10 | < LOQ |  |
| 2013 | < LOQ |  | < LOQ |  | < LOQ |  | 4.72 | ± 0.86 | < LOQ |  |
| 2015^#^ | 7.05 | ± 0.15 | 0.34 | ± 0.03 | < LOQ |  | 9.24 | ± 0.38 | < LOQ |  |

# **Supplemental References**

ECHA (2007): Difethialone: Assessment Report Product-type 14 (Rodenticides). Annex I. 21 June 2007. Rapporteur Member State: Norway. European Chemicals Agency

ECHA (2010a): Brodifacoum: Assessment Report Product-type 14 (Rodenticides). Annex I, 17 September 2009, revised 16 December 2010. Rapporteur Member State: Italy. European Chemicals Agency, Sweden

ECHA (2010b): Assessment Report Product-type 14 (Rodenticides). Annex I. 30 May 2008, revised 16 December 2010. Rapporteur Member State: Sweden. European Chemicals Agency

European_Chemicals_Bureau (2003): Technical Guidance Document on Risk Assessment (part II). In: European Commission IfHaCP (Hrsg.). EU, Ispra, Italy

Gomez-Canela C, Barata C, Lacorte S (2014): Occurrence, elimination, and risk of anticoagulant rodenticides and drugs during wastewater treatment. Environmental science and pollution research international 21, 7194-203
